# The impact of mitochondrial dysfunction on osteoarthritis cartilage: current insights and emerging mitochondria-targeted therapies

**DOI:** 10.1038/s41413-025-00460-x

**Published:** 2025-09-01

**Authors:** Siyuan Tan, Yujun Sun, Shixun Li, Haoyu Wu, Yue Ding

**Affiliations:** Department of Orthopedic Surgery, Sun Yat-Sen Memorial Hospital, Sun Yat-Sen University, Guangzhou, China

**Keywords:** Bone quality and biomechanics, Pathogenesis

## Abstract

Osteoarthritis (OA) is a degenerative joint disease associated with age, prominently marked by articular cartilage degradation. In OA cartilage, the pathological manifestations show elevated chondrocyte hypertrophy and apoptosis. The mitochondrion serves as key energy supporter in eukaryotic cells and is tightly linked to a myriad of diseases including OA. As age advances, mitochondrial function declines progressively, which leads to an imbalance in chondrocyte energy homeostasis, partially initiating the process of cartilage degeneration. Elevated oxidative stress, impaired mitophagy and mitochondrial dynamics jointly contribute to chondrocyte pathology, with mitochondrial DNA haplogroups, particularly haplogroup J, influencing OA progression. Therapeutic approaches directed at mitochondria have demonstrated remarkable efficacy in treating various diseases, with triphenylphosphonium (TPP) emerging as the most widely utilized molecule. Other strategies encompass Dequalinium (DQA), the Szeto-Schiller (SS) tetrapeptide family, the KLA peptide, and mitochondrial-penetrating peptides (MPP), etc. These molecules share common properties of lipophilicity and positive charge. Through various technological modifications, they are conjugated to nanocarriers, enabling targeted drug delivery to mitochondria. Therapeutic interventions targeting mitochondria offer a hopeful direction for OA treatment. In the future, mitochondria-targeted therapy is anticipated to improve the well-being of life for the majority of OA patients. This review summarizes the link between chondrocyte mitochondrial dysfunction and OA, as well as discusses promising mitochondria-targeted therapies and potential therapeutic compounds.

## Introduction

Osteoarthritis (OA) is a prevalent joint disease, marked by joint cartilage degeneration alongside secondary hyperostosis, and usually accompanied by pain and physical disability, consequently leading to work incapacitation and diminished quality of life.^1^ In addition to the pain associated with joint damage, patients with OA also presented allodynia and hyperalgesia around the joint due to nerve sensitization, which further reduces the quality of life.^2^ Prevalence studies consistently indicate a close association between OA and age, particularly evident post the age of 50.^3^ Other risk factors encompass obesity, metabolic diseases, joint malformation, mechanical load and trauma.^4^ With the increase of aged population in developed countries, the incidence of OA will significantly increase and become a burden for society. As the elderly population burgeons in developed countries, the incidence of OA is predictable to escalate significantly, constituting a substantial societal burden. According to an epidemiological investigation, prevalent cases of OA increased from 247.51 million to 527.81 million globally from 1990 to 2019, which has shown an amplification of 13.25%.^5^

Despite the fact that the pathological characteristics of OA involve impairment of the entire joint structure, including remodeling of subchondral bone, osteophyte formation, and synovial inflammation,^6^ the primary pathological hallmark remains cartilage damage. Articular cartilage is primarily composed of chondrocytes and extracellular matrix (ECM), which is comprised of water (up to 80% of the wet weight), type II collagen, hyaluronans, and other proteoglycans synthesized by chondrocytes.^7^ Due to the avascular property of cartilage, the chondrocytes rely on passive diffusion from the synovial fluid (SF) to obtain essential oxygen and nutrients. Under physiological conditions, articular chondrocytes highly depend on glycolysis for energy production.^8^ Nevertheless, mitochondrial oxidative phosphorylation still contributes about 25% of the ATP.^9^ Chondrocytes show metabolic flexibility, which supports cell survival under nutrient stress by enhancing mitochondrial respiration. However, in OA chondrocytes, this flexibility is weakened, manifested by declined mitochondrial respiration and enhanced anaerobic glycolysis.^10^

**Fig. 1:**
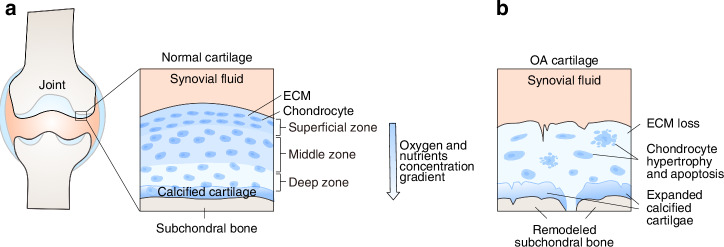
Articular cartilage structure and OA pathology. **a** Organization of articular cartilage. Articular cartilage mainly consists of chondrocytes and extracellular matrix (ECM). Oxygen and nutrients are delivered through the passive diffusion from the synovial fluid, thus forms a concentration gradient. **b** The microscopic pathological manifestations of OA cartilage show chondrocyte hypertrophy and apoptosis, as well as ECM loss and calcification

The microscopic pathological manifestations of OA cartilage show chondrocyte hypertrophy and apoptosis, as well as ECM loss and calcification^11,12^(Fig. 1). While chondrocytes normally maintain cartilage integrity ECM synthesis, this anabolic function is impaired in OA cartilage, and may even lead to cartilage degradation with direct involvement of chondrocytes.^13^ Apoptosis of chondrocytes leads to pericellular matrix degradation and the release of apoptotic bodies into chondrocyte lacunae and in the interterritorial space, promoting pathological calcification in OA cartilage.^14^ In physiological endochondral ossification, chondrocyte hypertrophy occurs as an intermediate stage between chondrocyte maturation and apoptosis. In OA joints, hypertrophic chondrocytes are present near the mineralized cartilage matrix and the surface lesions, with diminished synthesis of collagen II and aberrant expression of alkaline phosphatase (ALP). However, although the current mainstream view of chondrocyte hypertrophy as one of the characteristics of OA, this claim is still open to debate.^15^

In eukaryotic cells, the bilayer organelle mitochondrion supplies energy through peroxidation phosphorylation and has an independently inherited genome.^16^ The outer mitochondrial membrane (OMM), similar in composition to the plasma membrane, contains channels like voltage-dependent anion channel (VDAC) for small molecule transport, whereas the inner mitochondrial membrane (IMM) is protein-rich and contains negatively charged cardiolipin.^17^ Proteins comprising the respiratory chain reside on the IMM, bringing the tricarboxylic acid cycle in the matrix to convert ADP to ATP.^18^ Numerous studies have illustrated that mitochondrial dysfunction could lead to manifold diseases such as oncogenesis, neurodegenerative diseases, cardiovascular diseases, metabolic diseases, and musculoskeletal degenerative diseases, including OA.^19,20^

Currently, the organelle targeting treatment has gathered increasing interest among researchers. For most mitochondria targeting strategies, the molecules typically exhibit both lipophilicity and positive charge. The lipophilic nature facilitates insertion into the mitochondrial membrane via lipophilic affinity, while the positive charge interacts with the negative membrane potential and aids in translocating the molecule into the mitochondrial matrix.^21^ Since the mitochondrion is associated with multifarious diseases and its a pivotal role in cellular physiological processes, it is foreseeable that mitochondria-targeted drug delivery will emerge as a hotspot in biomedical research.

This review summarizes the link between chondrocyte mitochondrial dysfunction and OA, along with promising mitochondria targeting schemes and therapeutic compounds with potential implications for mitochondrial dysfunction. Our aim is to offer valuable perspectives into the advancement of mitochondria-targeted therapies for the treatment of OA.

## Mitochondria in OA

As previously noted, the hypoxic environment within the articular cavity traditionally suggests that chondrocytes primarily derive energy from glycolysis. The significance of mitochondria in chondrocyte degeneration has gained increasing recognition in the last 20 years. Present studies have emphasized the role of mitochondrial dysfunction in OA pathogenesis. Alterations in mitochondrial function induced by oxidative stress or mitochondrial DNA (mtDNA) heterogeneity have been implicated in chondrocyte apoptosis.^22^

### Mitochondrial oxidative stress and antioxidant system

Reactive oxygen species (ROS) refer to a broad category of oxygen-containing free radicals and peroxides that readily generate oxygen radicals within organisms, which includes hydroxyl radical (^•^OH), hydrogen peroxide (H_2_O_2_), superoxide anion (O_2_^•−^), and nitric oxide (NO), etc. Mitochondrial oxidative phosphorylation is one of the primary contributors of intracellular ROS.^23^ Oxidative stress refers to the imbalance between the generation of ROS and antioxidant defense, resulting in an elevated level of oxidation intermediates, ultimately culminating in mitochondrial dysfunction.

A heightened state of oxidative stress has been noted in patients with OA. A study has demonstrated that synovial cells in OA patients exhibit significant lipid peroxidation measured by the lipid peroxidation product malondialdehyde (MDA), possibly mediated by NO or other ROS produced by chondrocytes.^24^ Ostalowska et al. have found significantly increased antioxidant enzyme activity in knee osteoarthritis (KOA) patients, with the MDA level showing a rising trend (though not statistically significant). These findings suggest that elevated antioxidant enzyme levels may represent an adaptive response to increased ROS in SF, although the enzyme levels tend to decline as the disease progresses. They also found that the secondary OA exhibits heightened compensatory antioxidant responses and more pronounced oxidative tissue damage (lower SF viscosity), likely driven by trauma-induced inflammation, whereas primary OA reflects chronic adaptive mechanisms despite longer disease duration.^25^

**Fig. 2:**
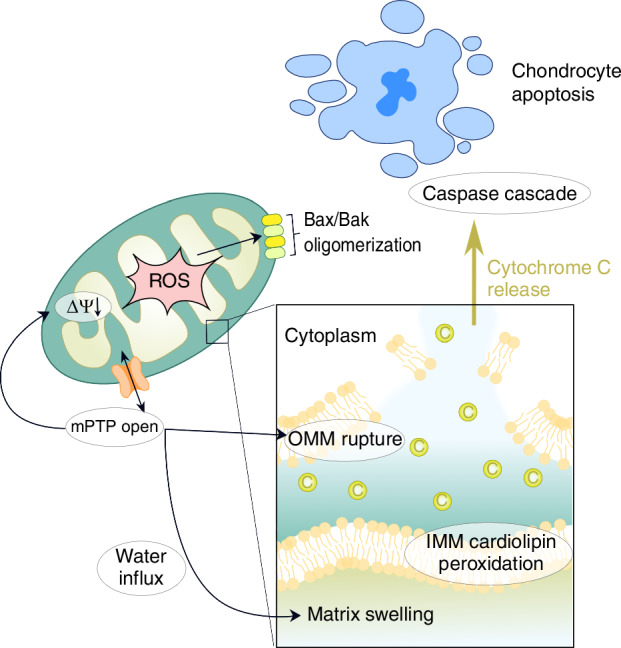
Excessive level of ROS promotes cytochrome c release via two distinct mechanisms: (1) MPT-dependent pathways, where ROS oxidize thiol groups on the adenine nucleotide translocator (ANT), inducing MPT, matrix swelling, and OMM rupture; and (2) MPT-independent pathways, involving cardiolipin peroxidation—which destabilizes cytochrome c binding to IMM—and subsequent release through OMM channels (e.g., VDAC) or Bax/Bak oligomerization. Cytochrome c then enters the cytoplasm and activates the caspase cascade to trigger chondrocyte apoptosis

Excessive level of ROS can induce mitochondrial damage by triggering mitochondrial permeability transition (MPT), membrane depolarization, IMM cardiolipin peroxidation, and cytochrome c release from both MPT dependent and independent pathway,^26^ which then enters the cytoplasm and activates the caspase cascade to trigger chondrocyte apoptosis,^27^ along with impaired mtDNA integrity and repair capabilities^28^(Fig. 2). This consequently instigates a cycle of sustained ROS production and release, fosters regional persistent oxidative stress and chronic inflammation,^29^ and eventually result in chondrocyte death.^30^ Moderate mechanical stress has been demonstrated to preserve mitochondrial function and effectively clearing ROS, thus mitigating IL-1β-induced chondrocyte apoptosis. Conversely, intense mechanical stress can induce severe mitochondrial dysfunction.^31^ Additionally, aging enhances mitochondrial-associated ROS production in chondrocytes, thereby exacerbating OA progression.^32^

Organisms possess antioxidants that counteract the effects of ROS, including superoxide dismutase (SOD), catalase (CAT) and glutathione (GSH), etc. Among these enzymes, SOD catalyzes the dismutation of O_2_^•−^ into H_2_O_2_ and O_2_, subsequently other antioxidants such as CAT converting H_2_O_2_ into H_2_O.^33^ The major O_2_^•−^ scavenger, the SOD family consists of three members: SOD1, SOD2 and SOD3. Among them, SOD2 is primarily localized in the mitochondria, while SOD1 predominantly resides in the cytoplasm, and SOD3 mainly exists in the ECM.

**Fig. 3:**
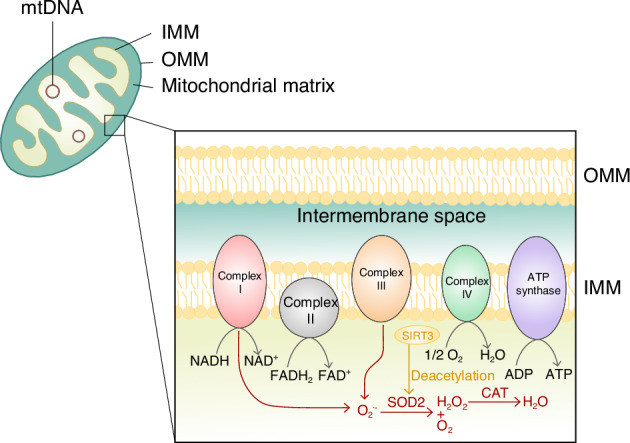
The structure of the mitochondrion. IMM and OMM are phospholipid bilayers surrounding mitochondrial matrix. The components of mitochondrial electron transport chain are located on IMM, including complexes I-IV and ATP synthase, among which the complexes I and III are the major source of O_2_^•−^. SOD2 catalyses the dismutation of O_2_^•−^ into H_2_O_2_ and O_2_, subsequently other antioxidants such as CAT converting H_2_O_2_ into H_2_O. The activity of SOD2 can be upregulated by the deacetylation of SIRT3. ΔΨ, mitochondrial membrane potential; mPTP, mitochondrial permeability transition pore; MPT, mitochondrial permeability transition; OMM, outer mitochondrial membrane; IMM, inner mitochondrial membrane

Studies have demonstrated that the SOD family is abundantly expressed in chondrocytes. However, in OA chondrocytes, there is a notable decrease in SOD expression, especially SOD2.^34,35^ Controversially, another research mentioned that serum SOD2 levels increased in patients with OA, indicating an elevated oxidative stress related with OA.^36^ Under oxidative stress, the expressions of both SOD1 and SOD2 are upregulated.^37^ Mechanical overload also reduced the expression and protein level of SOD2 gene in knee joint chondrocytes, which leads to excess production of mitochondrial superoxide, ultimately resulting in mitochondrial dysfunction and subsequent cartilage damage.^38^ The activity of SOD2 in chondrocytes declines with age,^32^ possibly due to reversible acetylation modification. However, this effect can be relieved by the deacetylation ability of SIRT3^39^ (Fig. 3).

SIRT3, a member of the sirtuin family, serves as a major deacetylase located in mitochondria that deacetylates various mitochondrial antioxidant enzymes, including isocitrate dehydrogenase (IDH2)^40^ and SOD2.^41^ Under oxidative stress, the SIRT3 expression is downregulated.^37^ Cytochrome c oxidase subunit 4 isoform 2 (COX4I2) is also a substrate of SIRT3, the deacetylation of which rescues maintain mitochondrial homeostasis and attenuates the progression of post-traumatic OA.^42^ Loss of SIRT3 impairs the function of antioxidant proteins by impeding mitochondrial aerobic respiration, leading to amassment of ROS and RNS and accelerating.^42^ The antioxidant ability of SIRT3 also depended on IDH2, which has been found to be able to defend cells against oxidative stress through elevated GSH levels.^43^

Nuclear factor (erythroid-derived 2)-like 2(Nrf2) is a transcription factor serves as a communication bridge between the nucleus and the mitochondria, responding to ROS-induced oxidative stress.^44^ The Nrf2 pathway is activated in response to oxidative stress, which is essential for mitigating mitochondrial ROS (mtROS) levels and preserving mitochondrial structural integrity.^45^ It has been proved that the kinases Mst1 and Mst2 detect ROS and preserve cellular redox by regulating the stability of Nrf2 in macrophages.^46^ In rat model of OA, the nuclear localization of Nrf2 enhanced by Transient Receptor Potential Vanilloid (TRPV)-1 activation via the Ca^2+^/calmodulin-dependent protein kinase II (CaMKII)/Nrf2 signaling pathway inhibited M1 macrophage polarization in synovium, thus attenuating OA progression.^47^

Heme oxygenase 1 (HO-1) is an iron-dependent antioxidant enzyme, with its expression and activity upregulated by Nrf2.^48^ In OA chondrocytes, the expression of HO-1 is elevated,^49^ but downregulated upon stimulation of pro-inflammatory factors such as TNF- α, IL-1β and IL-17.^50^ HO-1 plays a protective role by reducing ROS production, matrix metalloproteinases (MMPs) as well as pro-inflammatory factors in OA chondrocytes.^51^ In mouse models with HO-1 overexpressed, the level of SOD2 is upregulated and the chondrocytes apoptosis is suppressed.^52^ The activation of Nrf2/HO-1 axis suppresses OA development through downregulating NF-κB.^53^

Under oxidative stress conditions, Nrf2 has been demonstrated to upregulate the expression of PTEN-induced putative kinase 1(PINK1)^54^ and Parkin,^55^ ultimately inducing mitophagy.^56^ The activation of Nrf2/Parkin axis alleviates OA progress.^55^ Additionally, increased Nrf2 activity contributes to the degradation of Dynamin-Related Protein 1(Drp1), consequently inhibiting the fission of mitochondria.^57^ However, another study has demonstrated that Nrf2 over-expression activated ERK1/2,^50^ which in turn activates DRP1 in a pathologic condition.^58^ Nrf2 also modulates mitochondrial biogenesis via the activation of HO-1,^59^ potentially mediated by the production of carbon monoxide (CO).^60^ These findings indicate the association of oxidative stress with mitophagy and mitochondrial dynamics.

NOD-like receptor family, pyrin domain-containing 3 (NLRP3), serves as an intracellular sensor that detects a variety of pathogenic signals, triggering the assembly and activation of the NLRP3 inflammasome.^61^ Research has demonstrated that reduced level of ROS restrains the activation of NLRP3 inflammasome in vitro.^62^ Inhibition of TRPV4, a calcium-permeable cation channel, suppresses the polarization of M1 macrophages in synovium via the ROS/NLRP3 pathway, thereby delaying OA progression.^63^ Conversely, silencing Nrf2/HO-1 pathway activates the NLRP3 inflammasome,^64^ while elevated Nrf2 expression reduces mitochondrial ROS production, thereby inhibiting NLRP3 inflammasome activation,^65^ potentially contributing to the progression of OA.

Several other molecules have been reported to regulate the oxidative stress of mitochondria. Notably, sterol carrier protein 2 (SCP2) is highly expressed in human OA cartilage, where it is associated with the accumulation of lipid hydroperoxides (LPO). This accumulation leads to increased mitochondrial oxidative stress and subsequent ROS release. Inhibition of SCP2 can provide significant protection to mitochondria, reduce LPO levels, alleviate chondrocytes ferroptosis in vitro, consequently mitigate the progression of OA in rats.^66^ Kindlin-2 deficiency results in mice spontaneous OA and instability-induced OA lesions by exacerbating mitochondrial oxidative stress.^67^ The deletion of Krüppel-like factors 10/TGFβ inducible early gene-1 (Klf10/TIEG1) in mice can reduce ROS level in senescent mouse chondrocytes and restore mitochondrial membrane potential.^68^

### Mitophagy

Mitophagy is a process that selectively eliminates damaged or dysfunctional mitochondria through autophagy mechanism. Under normal physiological conditions, this process ensures mitochondrial homeostasis. It can be triggered by a variety of factors, including mechanical stress,^31^ hypoxia, oxidative stress^69^, and iron starvation.^70^ Mitophagy impairment leads to cell apoptosis and ECM degradation.^71^ Mitochondrial autophagy pathway is divided into Parkin-dependent pathway and Parkin-independent pathway.^71^

It has been proven that the Parkin-dependent pathway is triggered by significant mitochondrial depolarization induced by mitochondrial uncouplers^72^ or acute production of mtROS.^73^ Meanwhile, Parkin-independent pathway can be induced by hypoxia.^74^ However, few studies have clarified how chondrocytes choose between these two pathways.

Parkin-dependent pathway is induced by PINK1 and E3-ubiquitin ligase Parkin.^75^ There is a mitochondrial targeting sequence (MTS) attached to the N-terminus of PINK1, allowing PINK1 to be introduced into the IMM through translocases located in both of the OMM and the IMM in a mitochondrial transmembrane potential-dependent way. Then mitochondrial matrix processing peptidase and presenilin-associated rhomboid-like protein (PARL) cleave PINK1, maintaining a relatively low level of PINK1.^76,77^

When mitochondria sustain damage, abnormal mitochondrial transmembrane potential blocks the translocation of PINK1, therefore, PINK1 is bound to the translocase of the OMM. Parkin is attracted to the mitochondria from the cytoplasm and triggers ubiquitination of mitochondrial membrane proteins including VDAC1,^78^ mitofusin 1 (MFN1) and mitofusin 2(MFN2),^79^ subsequently facilitating the recruitment of p62/SQSTM1/sequestosome-1^78^ and microtubule-associated protein 1 light chain 3 (LC3).^80^ These effects impede the re-fusion of impaired and normal mitochondria, separating damaged mitochondria for subsequent incorporation into autophagosomes,^80^ which then fuse with lysosomes to degrade damaged mitochondria.^81^

Mild and sustained H_2_O_2_ stimulation can lead to Parkin-dependent mitophagy in vitro.^82^ In response to IL-1β stimulation, chondrocytes lacking Parkin expression exhibit compromised mitophagy, eventually resulting in mitochondrial dysfunction and elevated oxidative stress.^83^ Overexpression of Parkin can diminish mitochondrial ROS levels and chondrocyte apoptosis by clearing dysfunctional mitochondria.^84^ By comparison, a study conducted on a monosodium iodoacetate (MIA) -induced rat OA model revealed that deletion of Pink1 could mitigate cartilage damage.^85^

Activation of AMPK/SIRT3 pathway induces Parkin expression, parkin-dependent mitochondrial autophagy, and SOD2 activation, which coordinately alleviate mitochondrial stress.^86,87^ Through suppressing SIRT3 expression and consequently inhibiting Parkin-dependent mitophagy, IL-1β triggers chondrocytes apoptosis.^88^ α-ketoglutaric acid (α-KG) is a key metabolic intermediate in the mitochondrial tricarboxylic acid (TCA) cycle.^89^ It has been shown that α-KG level is lower in IL-1β-induced OA chondrocytes and human OA cartilage compared with normal cartilage. α-KG enhances the transcription of PINK1, Parki,n and other proteins, promoting mitochondrial autophagy and inhibits ROS production, thereby alleviating OA symptoms.^90^

Parkin-independent mitophagy pathway mainly relies on OMM proteins, which includes NIP3-like protein X/BNIP3-like protein(NIX/BNIP3L), BCL2 interacting protein 3 (BNIP3)^91^ and FUN14 domain protein 1 (FUNDC1).^74^ Skipping of ubiquitination, these proteins that can directly initiate mitophagy of the impaired mitochondria via the interaction between their LC3-interacting region (LIR) domain and LC3.^92^ The transcription of BNIP3 and NIX is positively modulated by hypoxia inducible factor 1 subunit α(HIF-1α).^93^ The expression of HIF-1α increases in OA cartilage, and promotes mitophagy to perform a protective effect on chondrocytes.^94^ Downregulation of Klf10 upregulates BNIP3, therefore promotes mitophagy.^68^ Knocking down proliferator-activated receptor-γ coactivator-1α (PGC-1α) can activate mitophagy by promoting the expression of BNIP3.^95^ ATAD3Bs is a novel mitotic receptor which contains a LIR motif that binds to LC3 and exacerbates Parkin-independent mitophagy induced by oxidative stress.^69^ Additionally, Mechanical stress strength-dependently promotes chondrocytes mitophagy.^31^

### Mitochondrial dynamics and biogenesis

Mitochondrial dynamics is defined as the dynamic equilibrium between the fusion and fission of mitochondria. This mechanism is essential to preserving mitochondrial quality and function.^96^ Dynamin-Related Protein 1(DRP1) is a GTPase, functioning as a key regulator of mitochondrial fission and can be upregulated by IL-1β.^97^ Suppression of DRP1-mediated mitochondrial fission blocks mitochondrial damage and chondrocyte apoptosis induced by IL-1β. ERK1/2 activates DRP1 in pathologic condition, the inhibition of which can suppress DRP1 activation, thereby mitigating mitochondrial network disruption and apoptosis in chondrocytes.^58^

TRPV4 is capable to activate DRP1 mitochondrial translocation via Ca^2+^ fluxion, leading to excessive mitochondrial fragmentation. In the anterior cruciate ligament transection (ACLT) mouse model, TRPV4 inhibition reversed cartilage degeneration mediated by DRP1.^98^ Chondrocyte-targeted delivery of Nrf2 inhibited the phosphorylation and mitochondrial translocation of DRP1, thereby averting mitochondrial fragmentation and dysfunction, consequently attenuating cartilaginous endplate degeneration in vivo.^99^ In OA mouse models, TANK-binding kinase 1 (TBK1) overexpression results in DRP1 Ser637 phosphorylation, inhibiting mitochondrial fragmentation and promoting the fusion of impaired mitochondria with autophagosomal membranes during mitophagy.^100^ In the presence of norepinephrine (NE) stimulation, mitochondrial dynamics in chondrocytes is enhanced through the upregulation of MFN1/2, Optic Atrophy Protein 1(OPA1) and DRP1.^101^

OPA1, along with MFN1/2, mediates mitochondrial fusion. MFN1/2 primarily regulate OMM fusion, whereas OPA1 predominantly manages IMM fusion.^102^ OPA1 is a GTPase, which is subject to the deacetylation and activation by SIRT3, thus regulating mitochondrial dynamics to preserve the integrity of mitochondrial network.^103^ Moderate mechanical stress fosters mitochondrial dynamics, preserving mitochondrial quality by upregulating MFN1/2 and OPA1 and facilitating the DRP1 translocation from cytoplasm to mitochondria.^31^ Conditional deletion of OPA1 leads to cartilage degeneration, eventually resulting in OA in aged mice.^104^

The expression of MFN2 is increased in both human and rat OA chondrocytes,^105^ along with chondrocytes stimulated by IL-1β.^106^ In the destabilization of the medial meniscus (DMM) rat model, Overexpression of MFN2 in articular cartilage accelerated the progression of OA, while knockdown of MFN2 mitigated cartilage degeneration.^107^ In contrast, another study has reported that overexpression of MFN2 enhances chondrocyte-specific gene expression and exhibits protective effects against OA.^108^ Stimulation of IL-1β downregulates the expression of MFN1.^97^ However, the relationship between MFN1 and OA chondrocytes has not been fully studied.

Adenosine monophosphate (AMP)-activated protein kinase (AMPK) is a pivotal regulator of energy homeostasis^109^ and is important for in mitochondrial biogenesis.^110^ The expression of AMPK is decreased in both mouse and human OA articular chondrocytes.^111,112^ Conversely, activation of AMPK signaling has been illustrated to protect chondrocytes from apoptosis.^113,114^ AMPK activator preserves mtDNA integrity and improves mitochondrial function in chondrocytes by reducing acetylation and upregulating SOD2 expression via the SIRT3 pathway, therefore attenuating aging-associated mouse KOA.^115^ Promoted AMPK signaling induces mitochondrial division, consequently increases the number of mitochondria in chondrocytes.^116^

AMPK regulates SIRT1, a NAD^+^-dependent deacetylase, with the important substrate PGC-1α.^117^ SIRT1, belonging to the same sirtuin family as SIRT3, predominantly localizes in the nucleus and the cytoplasm.^118^ The expression of SIRT1 is downregulated under various stresses known to induce apoptosis of human chondrocytes. The overexpression of SIRT1 counteracted the high glucose-induced upregulation of DRP1 and downregulation of MFN1, thereby diminishing mitochondrial fission.^119^ Inhibition of the AMPK /SIRT1/PGC-1α pathway reduces the expression of mitochondrial fusion proteins and suppresses mitochondrial function in chondrocytes, consequently exacerbating OA.^120,121^ In contrast, the activation of AMPK/SIRT1/PGC-1α pathway promotes mitochondrial biogenesis and fusion, thereby reducing oxidative stress in chondrocytes.^121,122^ The same effect in the spinal cord relieves OA pain.^123^

**Fig. 4:**
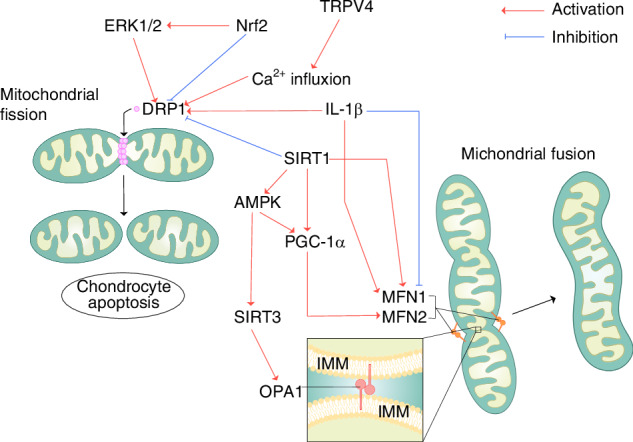
Summary of the key proteins and signaling pathways involved in mitochondrial dynamics. Mitochondrial fission is mainly mediated by DRP1, which binds to the OMM in a ring-like structure, and then the organelle divides into two separate mitochondria. OPA1, together with MFN1/2, mediates mitochondrial fusion, with MFN1/2 mainly regulating OMM fusion and OPA1 primarily controlling IMM fusion. Red arrows indicate activation or upregulation of pathway; while blue lines indicate repression or inhibition of the pathway. OMM, outer mitochondrial membrane; IMM, inner mitochondrial membrane; DRP1, Dynamin-Related Protein 1; MFN1 and MFN2, mitofusin 1 and mitofusin 2; OPA1, Optic Atrophy Protein 1

The key proteins and signaling pathways involved in mitochondrial dynamics are summarized in Fig. 4.

### mtDNA haplogroup

Mitochondria possess their own DNA, which is inherited independently from nuclear genomes. mtDNA is maternal inherited and exhibits a remarkably high mutation rate, allowing for continuous evolution through the accumulation of genetic variations.^22^ mtDNA haplogroup refers to a specific set of mtDNA polymorphisms with continent specificity.^124^ These haplogroups are intricately relevant to the pathogenesis, progression, and prognosis of OA.

The protective effects of mtDNA haplogroups can be manifested at the molecular level. mtDNA haplogroup J carriers have shown reduced consumption of oxygen and diminished production of ATP and ROS than non J variants,^125^ whereas haplogroup H carriers demonstrate higher mitochondrial oxidative damage, oxygen consumption and ROS production.^126^ In correspondence, haplogroup J cybrids display the same property, along with increased cell survival under oxidative stress.^127^ In vitro, chondrocytes from haplogroup J carriers demonstrate decreased NO production.^128^ In addition to influencing ROS production, mtDNA haplogroups have been illustrated to impact serum levels of antioxidant enzymes. Carriers of mtDNA haplogroup J exhibit a higher level of serum catalase compared to non-J carriers, while a trend of lower serum level of SOD2 has been found in carriers of haplogroup H.^36^

Besides, serum biomarkers relevant with OA are also affected. MMP3, also known as Stromelysin 1, can cause ECM cleavage^129^ while MMP13, or collagenase 3, is essential for the degradation of type II collagen, the primary component of superficial layers of cartilage.^130^ Both enzymes are upregulated with the progression of OA^13,131^ and make contributions to OA development by affecting joint cartilage ECM integrity.^132^ In OA patients, the serum level of MMP3 increases,^131,133^ and this change was more significant in carriers of haplogroup H. Additionally, a trend has been observed for haplogroup J carriers to have lower serum levels of MMP-13 than carriers of the mtDNA haplogroup H.^133^ Correspondingly, it has been observed that serum type II collagen in mtDNA haplogroup H carriers was more abundant, whereas haplogroup J carriers display lower levels.^134^ However, in haplogroup J cybrids, the expression of SIRT3 is initially lower than in H cybrids under moderate H_2_O_2_ stimulation. However, as the stimulation becomes severe, this difference disappears.^37^

The distinct impact of mtDNA haplogroups on OA is also clinically validated. Several European researchers have reported a reduced risk of KOA and hip OA in mtDNA haplogroup J carriers.^127,135,136^ Additionally, haplogroup T is linked to a decreased risk of KOA.^137^ Nevertheless, this effect may vary with regions and races.^138^ The association between mtDNA haplogroups and OA has also been described in Asian studies. Fang et al. has indicated that haplogroup B may serve as a protective factor against OA, while haplogroup G appears to have the opposite effect.^139^ This may because of the elevated mitochondrial oxidative phosphorylation in haplogroup G cybrids than in haplogroup B cybrids.^140^ Conversely, Koo et al. have suggested that mtDNA haplogroup B contributes to the onset of KOA.^141^

In radiography studies, knees of radiographic KOA patients carrying haplogroup J significantly decrease the risk of medium to large bone marrow lesions in the medial compartment.^142^ In patients with KOA, mtDNA haplogroup T was associated with the lowest increase in Kellgren-Lawrence grade, indicating a reduced risk of radiological progression.^143^ The TJ cluster of mtDNA haplogroups has revealed a milder radiographic OA progression, primarily driven by type T.^144^ In comparison, haplogroup Uk has been associated with rapid progression of OA.^145^(Table 1).

**Table 1:** Effects of mtDNA variants associated with OA

mtDNA haplogroup or cluster	Study subject	Effects associated with OA	Reference
haplogroup J	Spanish	decreased ROS production and mitochondrial respiration	^125^
	muscle samples of Spanish	decreased mitochondrial respiration	^126^
	143B. TK− Rho0 osteosarcoma cell cybrids	increased survival rate under oxidative stress	^127^
	human chondrocytes	decreased NO production	^128^
	serum from Spanish population	increased serum level of catalase	^36^
		decreased serum level of MMP13	^133^
	143B. TK− Rho0 osteosarcoma cell cybrids	decreased SIRT3 expression under moderate H_2_O_2_ stimulation	^37^
	CHECK and OAI cohorts	reduced risk of KOA	^127^
	Spanish	decreased risk of KOA	^135^
		less severe progression of KOA	
	Spanish	reduced risk of hip OA	^136^
	OAI cohorts	less severe progression of KOA	^142^
haplogroup H	muscle samples of Spanish	increased mitochondrial oxidative damage and ROS production	^125^
	143B. TK− Rho0 osteosarcoma cell cybrids	increased production of peroxide, peroxynitrite and mitochondrial anion superoxide	^127^
	serum from Spanish population	decreased serum level of SOD2	^36^
		increased serum levels of MMP3	^133^
		increased serum levels of the type II collagen	^134^
haplogroup T	British	decreased risk of KOA	^137^
	OAI cohorts	less severe progression of KOA	^143^
	south European		^145^
haplogroup U	Spanish	more severe progression of KOA	^135^
Haplogroup Uk	OAI cohorts	more severe progression of OA	^145^
haplogroup B	Chinese	decreased risk of OA	^139^
	Korean	increased risk of OA	^141^
haplogroup G	Chinese	increased risk of OA	^139^
		more severe progression of KOA	
	143B. TK− Rho0 osteosarcoma cell cybrids	increased mitochondrial oxidative phosphorylation	^140^

*OA* osteoarthritis, *OAI* osteoarthritis initiative, *CHECK* cohort hip and cohort knee, *ROS* Reactive oxygen species, *NO* nitric oxide, *MMP* matrix metalloproteinases, *KOA* knee osteoarthritis

## Treatments targeting on mitochondria

### Modified nanomaterials targeting on chondrocytes

In recent years, the polymer or lipid-based nanocarrier has emerged as a prominent drug delivery strategy. These nanoparticles are transported into cells through receptor-mediated endocytosis via caveolin-dependent, clathrin-dependent, or micropinocytosis pathways.^146^ Additionally, in order to ensure the rate of endocytosis, the optimal particle radius is preferably 25−30 nm.^147^

There have been reports regarding the use of modified nanocarriers for OA treatment. To target chondrocytes, the surface of these nanocarriers is typically modified with chondrocyte affinity peptides (CAP) or WYRGRL peptide. Another strategy takes advantage of the cationic charges of the nanocarriers to approach the chondrocyte ECM, which is composed of negative charged glycosaminoglycans,^148^ coinciding with the strategies of targeting mitochondria discussed later.

Primary chondrocytes-derived exosomes cultured under normal conditions have been demonstrated to ameliorate mitochondrial dysfunction and impede OA progression.^149^ Transplantation of mitochondria derived from bone marrow mesenchymal stem cells has been shown to promote mitochondrial biogenesis.^106^ Kim et al. researchers have designed fusogenic capsules synthesized by a neutral lipid (1,2-distearoyl-sn-glycero-3-phosphoethanolamine, DSPE) and a cationic lipid (1,2-dioleoyl-3-trimethylammonium-propane, DOTAP) to effectively encapsule and transfer mitochondria from mesenchymal stem cells to chondrocytes, leading to reduction in the expression of inflammatory cytokines and MMP13, while promoting cartilage regeneration. Besides, they observed that the encapsulation efficiency increased in proportion to the amount of DOTAP, indicating that cationic substance exhibit affinity with anionic mitochondria.^150^ Chen et al. have constructed the cartilage-targeted nanomicelles modified with the WYRGRL peptide, which were developed to deliver Pioglitazone. The nanomicelles reduced intracellular ROS levels and restored the mitochondrial membrane potential, eventually inhibited H_2_O_2_-induced chondrocyte death and delayed OA progression.^151^

### Mitochondria-targeting strategies

Constructing nanoparticles through coating drugs with lipids and subsequently modifying their surface with mitochondria-targeting molecules has proven to be a viable approach. Examples of these molecules include triphenylphosphonium (TPP), Dequalinium (DQA), Szeto-Schiller (SS) tetra-peptide family, the KLA peptide, mitochondrial penetrating peptides (MPP), etc.^152^ (Table 2).

**Table 2:** Overview of current strategies targeting mitochondrial function and their applications

Modification Strategy	Active Molecule	Functions of molecules	Methods of Modification	Carrier system	Therapeutic agents	Study subject	Reference
modification with cations	TPP	mitochondrial targeting	TPP-PEG-DSPE	liposomes	black phosphorus, calcium peroxide	RM-1 cells	^217^
			TPP-PDL	exosomes	circRNA mSCAR	macrophages	^158^
			TPP-decorated amphiphilic cationic peptides	endosomal pH-responsive polymer	circRNA-expressing vector	liver fibroblasts	^218^
			direct combination	mesoporous silica nanoparticle-amidated	EGTA (calcium chelator)	bone marrow-derived macrophages	^159^
			direct combination	-	ortho-carbonyl hydroquinones	human platelets	^219^
	DQA	mitochondrial targeting, antibacterial and anticancer activity	insertion	liposomes	DQA	U373-MG cells	^162^
			emusification	nanoemulsion based on castor oil and water	DQA, α-tocopherol succinate	HeLa cells	^220^
			thin-film hydration	Pluronic® F68	curcumin/n-acetylcysteine/deferoxamine	SH-SY5Y cell	^221^
			methyl acrylate linker	glycol chitosan	curcumin	HeLa cells	^163^
modification with peptides	SS peptides	mitochondrial targeting, antioxidant activity	SS31-sulfhydryl	Mn3O4@PDA@Pd	Mn3O4, Pd	rat chondrocytes	^176^
			direct combination	pH/ROS dually responsive CsA nanomicells-loaded injectable hydrogel	cyclosporine A	H9C2 cells	^222^
			direct combination	-	Tiron	human lung microvascular endothelial cells	^177^
	MPP	mitochondrial targeting	direct combination	-	pyrrole-imidazole polyamides, chlorambucil	HeLa cells	^180^
			direct combination	N-(2-hydroxypropyl) methacrylamide copolymer	DOX	4T1 cells	^181^
	KLA peptides	mitochondrial targeting, induction of apoptosis	polymerization at the emulsion interface	polymeric dual-drug nanoparticle	OxPt-CDM	A549 cells	^183^
			direct combination	CGGGKLVFF-tk-PEG-PVA	nanofibers fibrous structures	HeLa cells	^184^
modification with other molecules	DOX-PEG-lipid	mitochondrial targeting, anticancer activity	DSPE-PEG_2000_-DOX	mesoporous silicon-coated gold nanorods	BNN6	4T1 cells	^187^
	pyruvate	specifically bind with the MCTs on the outer surface of mitochondria	formation during hydrothermal process	lipid membrane-coated silica-carbon	DOX/paclitaxel/irinotecan	NCI/RES-ADR cells	^189^
compounds that naturally target on mitochondria	DMY	activate SIRT3	-	hyaluronic acid methacrylate-gelatin methacrylate-PBA	DMY	mouse chondrocytes	^191^
	UA	promote mitophagy	-	WYRGRL modified liposomes-methacrylate hyaluronic acid hydrogel microspheres	UA	rat chondrocytes	^193^

*TPP* triphenylphosphonium, *DQA* Dequalinium, *SS* peptides, Szeto-Schiller tetra-peptide family, *MPP* mitochondrial penetrating peptides, *DOX* Doxorubicin, *PEG* polyethylene glycol, *DMY* Dihydromyricetin, *UA* Urolithin A, *MCT* monocarboxylate transporters, *DSPE* 1,2-distearoyl-sn-glycero-3-phosphoethanolamine, *PDL* poly-D-lysine

#### Cations for modification

For most mitochondria-targeting cations, the molecules share two common properties: lipophilicity and positive charge. The former enables insertion into the mitochondrial membrane via lipophilic affinity, while the latter capacitate translocation into the mitochondrial matrix depending on the negative membrane potential, which can reach up to -180 mV.^21,153^

Among cations, TPP has been most extensively applied for modification to target mitochondria. TPP is a lipophilic cation featuring a phosphorus atom surrounded by three hydrophobic phenyl groups.^21^ Its ability to aggregate in mitochondria has already been discovered since the 1960s, the mechanism of which has been thoroughly explored.^154–156^ According to the Nernst equation, at physiological temperatures, for every 60 mV increase in membrane potential, the accumulation of TPP within mitochondria will increase by a factor of 10. Given that the mitochondrial membrane potential typically ranges from 140 to 180 mV, the TPP cation is able to accumulate several hundred-fold within the mitochondrial matrix, presenting a highly efficient targeting ability.^154^ During the last 20 years, a series of mitochondria-targeted antioxidants have been developed through the covalent attachment of the TPP cation to various antioxidant molecules, including derivatives of ubiquinol (MitoQ) and alpha-tocopherol (MitoVit E), etc.^154,157^

Recently, increasingly numerous studies have combined TPP with nanocarriers to target mitochondria for drug delivery. In another research, modified exosomes coated with circRNA mSCAR (a mitochondrial circular RNA) with TPP- poly-D-lysine (PDL) were able to target macrophages mitochondria, thereby reducing the level of mtROS, facilitating the polarization of macrophages towards M2 phenotype and alleviating sepsis in mice.^158^ In another study, nanoparticles modified with TPP were synthesized to selectively target bone marrow-derived macrophages mitochondria. These nanoparticles inhibited production of mtROS, thus performs protective effect against OA.^159^

DQA is a lipophilic dication, consisting of two cationic quinolinium moieties connected by a 10-carbon alkyl chain.^21^ The chloride of DQA has antibacterial and anticancer activity.^160,161^ Bae et al. constructed nanoparticles composed of DQA/DOTAP/ 1,2-dioleoyl-sn-glycero-3-phosphoethanolamine (DOPE), which enhanced ROS production and impaired mitochondrial membrane potential, eventually inducing cell apoptosis.^162^ In another study, for cancer therapy, researchers synthesized an amphiphilic polymeric nanoparticle using glycol chitosan, with DQA serving as both the mitochondria-targeting moiety and the lipophilic component.^163^ However, due to the cytotoxicity of DQA, which inhibits the production of mitochondrial ATP and thus inhibits cell growth,^164^ it may be difficult to apply DQA in the field of cell repairment.

In addition to these two cations, rhodamine derivatives are also able to selectively accumulate in mitochondria.^165^ The targeting mechanism is similar to the two cations mentioned above, but because rhodamine has one less positive charge than the two positive charges of DQA, its targeting ability and therapeutic efficacy are considered to be not as good.^164^ Rhodamine is often applied as dyes for fluorescent probes and biomolecular tracer.^166,167^ At higher concentration, it performs cytotoxity towards cancer cells.^168^ Rhodamine 19 derivatives can act as mild mitochondria-targeted cationic uncouplers, causing a limited decrease in mitochondrial membrane potential.^169^ Consequently, ATP production would decrease, but ROS production would also decline.^170^ However, researches on rhodamine derivatives modification for mitochondrial-targeted drug delivery are extremely rare.

#### Peptides for modification

The SS-peptides are small, water-soluble tetrapeptides characterized by a structural motif of alternating basic amino acids and aromatic residues. This structural feature allows the SS peptides to selectively accumulated on IMM depending on mitochondrial membrane potential.^171^ It has been found that SS03 could achieve a 100-fold concentration in mitochondria after incubated with mouse liver mitochondria.^172^ Nevertheless, their ability to pass into the mitochondrial matrix is relatively insufficient.^172^ The components of the SS peptides include tyrosine (Tyr), dimethyltyrosine (Dmt), arginine (Arg), phenylalanine (Phe), and lysine (Lys) residues, among which Tyr and Dmt perform antioxidant activity and scavenge ROS.^21^

The mechanism by which SS peptides pass through OMM is poorly reported, but given that the composition of OMM is similar to the plasma membrane, there may be some reference. Take SS02 as an example, prior to the discovery of its mitochondrial targeting ability, SS02 was considered an opioid analog. It has been demonstrated that there is an energy-independent way for the cellular uptake of SS02.^173^

Treatment with SS31 alone could reserve mitochondrial function through Sirt3/PGC-1α signaling pathway^174^ and upregulate OPA1 via Sirt3.^175^ Li et al. have constructed a nanozyme called Mn_3_O_4_@PDA@Pd-SS31, which targeted mitochondria via SS31 modification. This nanozyme simulated SOD activity, effectively clearing chondrocyte mtROS, thereby reserving mitochondrial dysfunction, promoting mitophagy, and alleviating OA progression.^176^ Hui Wang et al. used the combination of SS peptides and the antioxidant Tiron to achieve a significant diminishing of LPS-mediated mtROS production in mouse LPS model of acute lung injury.^177^

Inspired by SS peptides, Mitochondrial penetrating peptides (MPP) were primarily synthesized by Horton et al. MPP contain four or eight residues, forming a motif of alternating cationic and hydrophobic residues comparable similar to SS peptides, with Lys and Arg providing positive charge while Phe and cyclohexylalanine residues imparting lipophilicity.^178^ Yousif et al have reported that rather than endocytosis, MPP seems to cross the plasma membrane through direct uptake, and relys on a balanced, dispersed distribution of cationic charge and hydrophobicity to traverse mitochondrial membranes. In addition, successful cargo delivery requires a logP ≥ -2.5 of the drug (logP: The logarithm of a compound’s partition coefficient between octanol and water, indicating its lipophilicity and membrane permeability potential).^179^

A compound composing pyrrole-imidazole polyamides, MPP and chlorambucil has been discovered to be capable of selectively modulating mtDNA mutations.^180^ Yang et al. have designed a MPP-modified Doxorubicin-loaded N-(2-hydroxypropyl) methacrylamide (HPMA) copolymer conjugates to target mitochondria, aiming to address mitochondrial dysfunction and achieve anticancer effects.^181^

The KLA peptides with amino acid sequence KLAKLAKKLAKLAK also possess mitochondrial targeting ability. Primarily, they were employed as disruptors of the mitochondrial membrane to induce cell apoptosis,^182^ thus making them mainly applicable in tumor therapy due to their cytotoxic properties. Yang et al. constructed a polymeric dual-drug nanoparticle that integrates an oxaliplatin derivate with the mitochondria-targeting peptide FKLAK. This innovative nanoparticle could disrupt mitochondrial membrane potential and inhibit ATP-dependent processes, such as drug efflux and DNA damage repair in tumor cells, resulting in enhanced anticancer activity.^183^ Cheng et al. utilized the KLA peptide to modify nanoparticles equipped with a thioketal linker that could respond to elevated levels of ROS around mitochondria, subsequently transform into the fibrous structures, and demonstrated multivalent cooperative interactions with mitochondria.^184^

#### Other modification methods

Doxorubicin (DOX) has been extensively used as an anticancer drug in clinic. However, it performs cardiotoxic effects, with mitochondrial membrane as the major target of cellular toxicity.^185^ Xi et al. initiatively found that a straightforward amphiphilic modification, realized by connecting lipids to DOX through a PEG linker, could enable its selective accumulation in tumor cell mitochondria in vivo.^186^ This strategy has been used by Wang et al. to construct a mesoporous silicon-coated gold nanorods modified with DSPE-PEG_2000_-DOX, the nanoparticle played the role of the carrier of NO prodrug (BNN6) and achieved the mitochondrial site-specific release of NO.^187^

The physiological process of mitochondrial respiration can also be exploited as a target. Pyruvate, an organic compound that participates in the tricarboxylic acid cycle, can be specifically transported into mitochondria by the monocarboxylate transporters (MCTs) on the OMM.^188^ Wang et al. used pyruvate to modify a silica-carbon hybrid nanoparticle coated with lipid membrane, thereby specifically producing ROS in mitochondria and targeting DOX to mitochondria.^189^

### Nanomaterials naturally targeting on mitochondria

Despite modified compounds, there exist other organic molecules that naturally targeting mitochondria. Dihydromyricetin (DMY) is a natural agonist of SIRT3,^190^ which is a deacetylase localized in mitochondria as mentioned before. Xia et al. used microfluidic technology to achieve the formation of a molecule termed DMY@HGP, which consisted of gelatin methacrylate and benzenediboronic acid (PBA) anchored to hyaluronic acid methacrylate. Given PBA’s sensitivity to ROS, intra-articular injection of DMY@HGP ROS-responsively restored the endogenous balance between mitochondrial apoptosis and mitophagy, consequently ameliorating cartilage wear and subchondral osteosclerosis in mice with an OA model induced by trauma.^191^

Urolithin A(UA) is a natural compound known to activate mitophagy and promote mitochondrial respiration in primary chondrocytes from both healthy donors and OA patients. This activity contributed to the mitigation of cartilage degeneration in a mouse OA model.^192^ Chen et al. modified chondrophilic peptide (WYRGRL) on the surface of UA-carrying liposomes by microfluidic technology. These liposomes were then integrated into methacrylate hyaluronic acid hydrogel microspheres, which were capable of targeting chondrocytes and selectively remove subcellular dysfunction mitochondria.^193^

### Other medicines affecting chondrocytes mitochondria

There are several medicals illustrated to affect mitochondria, either by diminishing oxidative stress or by activating signaling pathways on mitochondrial dynamics.

Pretreatment with Angelica sinensis polysaccharide (ASP) has been demonstrated to notably activate SOD2 in tert butyl hydroperoxide (TBHP) -cultured rat chondrocytes, effectively clearing ROS and promoting mitochondrial metabolism.^194^ In addition, ASP exhibits protective effects in chondrocytes against oxidative stress-induced apoptosis mediated by mitochondrial signaling pathway.^195^ Prussian Blue nanoparticles (PBNPs) exhibit antioxidant activity. Zhou et al. discovered that PBNPs alleviated intracellular oxidative stress by stabilizing SOD1, thereby preserving mitochondrial structure, enhancing antioxidant capacity, and ultimately rescuing ROS-induced intervertebral disc degeneration in a rat model.^196^ Astaxanthin (ATX) is a natural ketocarotenoid pigment. Wang et al. have illustrated that ATX was able to restore GSH levels, clear ROS, reserve mitochondrial membrane potential, and structure damage in chondrocytes under the stimulation of IL-1β.^197^

Metformin has been demonstrated to upregulate both AMPK and LC3 expression in articular cartilage tissue,^114,198^ and act as an activator of Parkin-dependent mitophagy via the SIRT3 signaling pathway, combating IL-1β-induced oxidative stress in chondrocytes.^87^ Administration of metformin has significantly reduced cartilage degradation in DMM mouse model.^114^ Gastrodin (GAS), derived from *Gastrodia elata*, along with Irisin, a soluble peptide upregulated by the PGC-1α signaling pathway, and Mitochonic acid-5, a mitochondrial homing drug, all activate the SIRT3/Parkin pathway, thus enhancing mitochondrial membrane potential and promoting mitophagy.^88,97,199^ Koumine, an alkaloid extracted from Gelsemium elegans,^200^ alleviates chondrocyte inflammation by activating Parkin-mediated mitophagy, consequently slowing down the progression of OA.^201^

Trehalose, a natural disaccharide, demonstrates efficacy in mitigating mitochondrial membrane potential impairment induced by oxidative stress, ATP depletion and DRP1 translocation into the mitochondria.^202^ Nodakenin (Nod) is one of the main active components in Radix Angelicae biseratae, a traditional Chinese medicine for OA treatment. Research has demonstrated that Nod inhibited DRP1 phosphorylation, thus suppressing ROS generation via DRP1-dependent mitochondrial fission in LPS-stimulated chondrocytes in vitro and attenuated cartilage degradation and inflammation in a mouse OA model.^203^ Platelet-rich plasma (PRP) intraarticular injection, commonly used for OA treatment,^204^ has been proven to inhibit mitochondrial damage mediated by LPS through diminishing ROS production, inhibiting DRP1 expression, and upregulating MFN1 expression, eventually repairing mitochondrial function.^205^

## Challenges and future perspectives

Although mitochondrial-targeted delivery of drugs can reduce toxicity and increase therapeutic effectiveness, several challenges remain to be addressed. There are some researches on mitochondrial-targeted delivery of drugs for OA treatments, but few have been tested in clinical settings. To achieve the goal of OA treatment, the drugs or nanocarriers should possess key properties, including chondrocyte or synoviocyte selectivity, activity of an antioxidant or interaction with signaling pathways such as SIRT3/Parkin and AMPK /SIRT1/PGC-1α, etc. By incorporating these properties, the therapeutic compound can restore mitochondrial function, reduce oxidative stress, and ultimately slow down the progression of OA. Therefore, mitochondria-targeting molecules should be carefully selected, as DQA, rhodamine derivatives, KLA peptides and DOX-lipid mentioned above are cytotoxic and more suitable for anticancer therapy. The SS peptides, despite their antioxidant activity, the relatively weaker ability to enter the mitochondrial matrix may limit their application.

There are many options for therapeutic compounds delivered to the mitochondria. In addition to the drugs that interact with mitochondria described above, nanoenzyme and RNA are also promising. This review summarizes the mitochondria-related protein changes and signaling pathways in OA, which may be potential targets to search for appropriate compounds. For example, miR-30 families have been demonstrated to interact with DRP1 in several researches,^206–208^ and it has been confirmed that the expression of miR-30b is upregulated in OA chondrocytes.^209^ MnO_2_ as a nanoenzyme has been illustrated to simulate SOD activity and protect cartilage from oxidative stress,^210,211^ it can also modulate macrophage phenotype and reduce inflammation.^212,213^

In addition to regulating mitochondria-related proteins and clearing mtROS, targeting mtDNA provides a new idea for OA therapy to improve cell metabolism and anti-inflammatory from the root, especially in the early intervention potential. Mitochondrial replacement technology (MRT) has been reported as a method to prevent severe mitochondrial diseases in newborns with maternal genetic risks by transplanting the nuclear genome from an affected egg to an enucleated donor egg. However, its application between donors and recipients with different haplogroups is not recommended to avoid reversion to affected mtDNA.^214^ While there has been research suggesting that eggs with random haplogroups can serve as donors,^215^ the suitability of MRT for preventing degenerative diseases like OA and its ethical issues are still open to debate. RNA-free DddA-derived cytosine base editors (DdCBEs), a base editor specifically converting C-G to T-A in human mtDNA reported by Mok et al.^216^ may serve as a potential OA prevention method by precise editing of mtDNA. Nevertheless, identifying precise sequence targets for editing and assessing potential side effects remains challenging.

Besides, bare therapeutic compounds may be degraded or inhibited by biomolecules in the cytoplasm. In consequence, nanocarriers can be a promising way to handle this issue. For clinical application, the ideal nanocarrier should have the characteristics of self-assembly, ROS response, and easy preservation. Due to the avascular nature of the joint cavity, the clearance of nanocarriers mainly depends on lymphatic drainage, and larger particles may result in delayed clearance.

## Conclusion

In this Review, we have outlined the molecular processes pertaining to the interplay between chondrocyte mitochondria and the onset and progression of OA from diverse perspectives. Recently, organelle targeting has emerged as a promising method for precision drug delivery. Molecular modifications stand out as the primary strategy for mitochondrial targeting, including cations and peptides modification. The common principle of the two methods hinges on taking advantage of the negative mitochondrial membrane potential to attract the positively charged molecules. Additionally, we have highlighted some drugs capable of impacting mitochondria via pathways involving oxidative stress, mitophagy, or mitochondrial dynamics.

In conclusion, mitochondria exert a significant influence on the pathogenesis and progression of OA. Therapeutic strategies targeting mitochondria represent a novel direction in OA treatments. Further exploration of mitochondrial dysfunction’s role in OA pathogenesis and the mechanisms underlying mitochondria-targeted therapies holds the potential to inspire more efficacious treatments for OA patients. In the future, mitochondria targeted therapy is expected to bring better life quality to the majority of OA patients.
